# Targetome Analysis Revealed Involvement of MiR-126 in Neurotrophin Signaling Pathway: A Possible Role in Prevention of Glioma Development

**DOI:** 10.22074/cellj.2018.4901

**Published:** 2018-03-18

**Authors:** Maedeh Rouigari, Moein Dehbashi, Kamran Ghaedi, Meraj Pourhossein

**Affiliations:** 1 Isfahan Neuroscience Research Center (INRC), Alzahra Hospital, Isfahan University of Medical Sciences, Isfahan, Iran; 2 Genetics Division, Department of Biology, Faculty of Sciences, University of Isfahan, Isfahan, Iran; 3 Cell and Molecular Biology Division, Department of Biology, Faculty of Sciences, University of Isfahan, Isfahan, Iran; 4 Department of Genetics and Molecular Biology, School of Medicine, Isfahan University of Medical Sciences Isfahan, Iran; 5 Department of Food Science and Technology, Food Security Research Center, School of Nutrition and Food Science, Isfahan, Iran

**Keywords:** EGFL7, Glioma, IRS-1, MiR-126, Neurotrophin

## Abstract

**Objective:**

For the first time, we used molecular signaling pathway enrichment analysis to determine possible involvement of miR-126 and IRS-1 in neurotrophin pathway.

**Materials and Methods:**

In this prospective study, validated and predicted targets (targetome) of miR-126 were collected following searching miRtarbase (*http://mirtarbase.mbc.nctu.edu.tw/*) and miRWalk 2.0 databases, respectively. Then, approximate expression of miR-126 targeting in Glioma tissue was examined using UniGene database (*http://www.ncbi. nlm.nih.gov/unigene*). In silico molecular pathway enrichment analysis was carried out by DAVID 6.7 database (*http://david. abcc.ncifcrf.gov/*) to explore which signaling pathway is related to miR-126 targeting and how miR-126 attributes to glioma development.

**Results:**

MiR-126 exerts a variety of functions in cancer pathogenesis via suppression of expression of target gene including *PI3K, KRAS, EGFL7, IRS-1* and *VEGF*. Our bioinformatic studies implementing DAVID database, showed the involvement of miR-126 target genes in several signaling pathways including cancer pathogenesis, neurotrophin functions, Glioma formation, insulin function, focal adhesion production, chemokine synthesis and secretion and regulation of the actin cytoskeleton.

**Conclusion:**

Taken together, we concluded that miR-126 enhances the formation of glioma cancer stem cell probably via down regulation of IRS-1 in neurotrophin signaling pathway.

## Introduction

MicroRNAs (miRNAs) are defined as endogenous small and non-coding RNAs that are approximately 18-24 nucleotides in length and play a significant role in the regulation of gene expression (1). These non-coding RNAs are negative regulators of gene expression (2). MiRNAs may have roles not only as oncogenes but also as tumor suppressors, further suggesting them as therapeutic targets (3). The interaction between miRNAs and 3´-nontranslatable regions of target mRNAs by complementarity, triggers mRNA degradation or prevents its translation (2, 4). As of today, more than 2000 different miRNAs have been characterized (2). MiRNAs could be considered as a valuable diagnosis or prognosis biomarker to predict the likely outcome of certain diseases such as cancer (5). 

**Fig.1:**
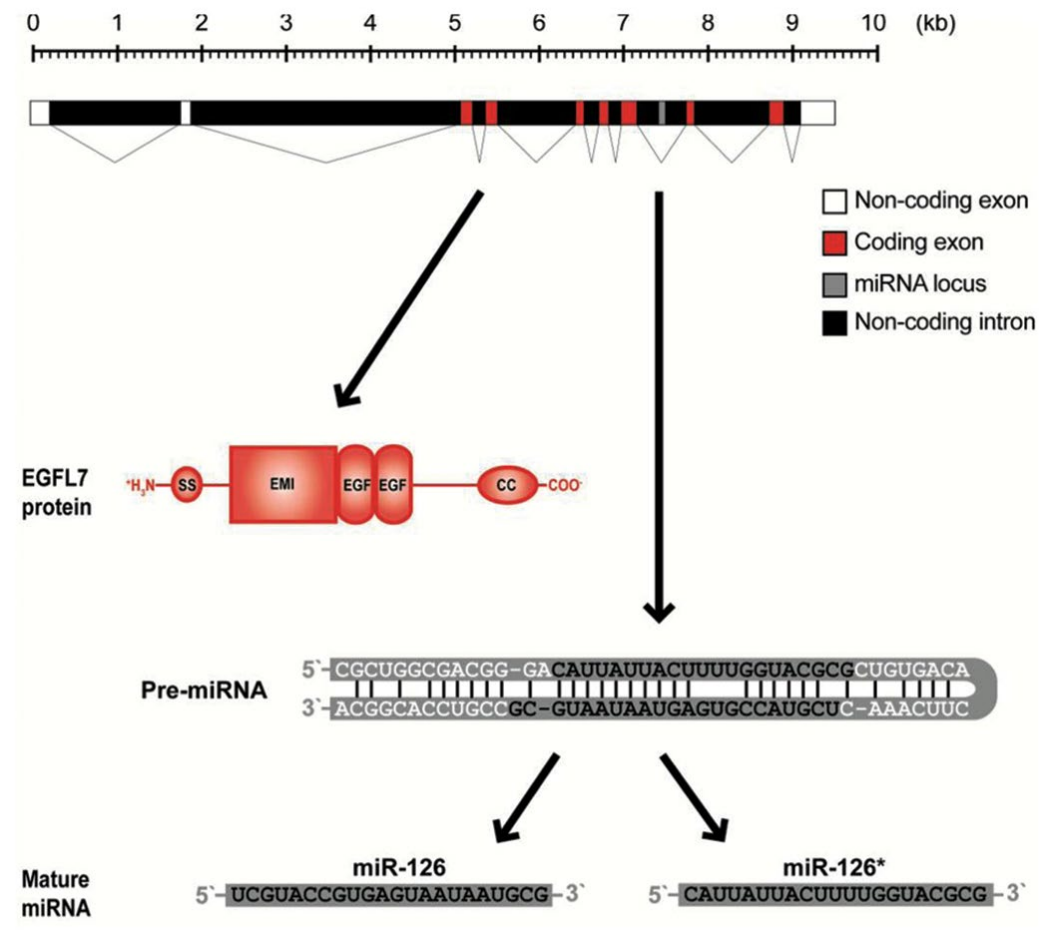
Structural organization and products of the EGFL7 gene. The EGFL7 gene contain 10 exons, but only exons 3-9 encode for the EGFL7 protein, which exhibits a modular structure with an N-terminal signal secretion peptide (SS) followed by an Emilin-like domain (EMI), two epidermal growth factor-like domains (EGF), and a coiled-coil (CC) region. A premiRNA structure is placed in intron 7 of the EGFL7 gene from which miR126 and miR-126* originate (8).

Glioma as one of the main central nervous system (CNS)-related cancers, comprises nearly 30% of all brain and CNS tumors and 80% of all malignant brain tumors (6). miR-126 is found on chromosome 9 within intron 7 of epidermal growth factor like domain 7 (*EGFL7*) gene (Fig .1) (7). miR-126 is one of the most important miRNAs that has significant roles in cellular biology, including cancer biology. Schmidt and colleagues showed that miR-126 is mainly involved in angiogenesis and inflammation, and briefly reviewed that miR-126 may play crucial roles in several human cancers (8). 

In this regard, a number of miR-126 target genes has already been characterized. *EGFL7* is the host gene of miR-126, which is also the major target of miR 126. Transcription of *EGFL7* and miR-126 occurs simultaneously and mature miR-126 matches with a complementary sequence within the host gene *EGFL7*, causing a decrease in EGFL7 protein levels and activating a negative feedback mechanism. EGFL7 affects cell migration pathways by interfing with tissue invasion and angiogenesis. Negative feedback mechanism of miR-126 shows that one of the main functions of miR-126 is regulating EGFL7 protein formation, leading to reduction of angiogenesis and cell migration (9). 

Another target of miR-126 is insulin receptor substrate-1 (IRS-1) as upregulation of miR-126 can significantly inhibit the expression of IRS-1 (10). IRS-1 is able to modify PI3K/Akt signalling through neurotrophin pathway which is one of the major pathways involved in neural cell differentiation (11). Furthermore, V-Ki-ras2 Kirsten rat sarcoma viral oncogene (KRAS) is considered another target gene of miR-126. KRAS is involved in cellular differentiation via PI3K/Akt pathway (12). Interestingly, Ras and PI3K proteins are associated with the glioma cancer cells formation through Notch, Hedgehog (Hh), Wnt/betacatenin, and focal adhesion pathways (13). Therefore, upregulation of miR-126 may suppress IRS-1 and subsequently trigger the formation of glioma cancer stem cell (14). 

Cancer progression needs permanent formation of blood vessels to nourish the cancer cells. Thus, controlling this factor is of immense importance in inhibition of cancer progression (15). Furthermore, miR-126 can affect many cellular mechanisms involved in cancer pathogenesis via suppressing translation of numerous validated target gene such as *PI3K, KRAS, EGFL7, CRK, ADAM9, HOXA9, IRS-1, SOX-2, SLC7A5* and *VEGF* in colorectal, gastric, oesophageal, oral, pancreatic, liver, thyroid, breast, cervical, ovarian, prostate, bladder, renal, and lung cancers as well as melanoma, osteosarcoma and leukemia (16). We here used molecular signaling pathway enrichment analysis to determine possible associations between miR-126 and IRS1 within neurotrophin pathway, for the first time.

## Materials and Methods

In this prospective study, all miRNA-mRNA prediction analyses were conducted by miRWalk 2.0 database (zmf.umm.uni-heidelberg.de/apps/zmf/ mirwalk2/) which is an integrative mRNA-miRNA prediction database (17). In order to find validated interactions, we implemented miRtarbase database (*http://mirtarbase.mbc.nctu.edu.tw/*) which provides experimentally validated mRNA-miRNA interactions from all previously performed studies (18).

Then, approximate expression of miR-126 targeting in glioma tissue was examined using UniGene database (*http://www.ncbi.nlm.nih.gov/unigene*). Finally, in silico molecular pathway enrichment analysis was carried out by DAVID 6.7 database (*http://david.abcc. ncifcrf.gov/*) (19) to explore which signaling pathway is related to miR-126 targetome and how miR-126 attributes to the formation of glioma. This database utilized the P values associated with each annotation terms inside each cluster are exactly the same meaning/ values as those (Fisher’s Exact/EASE Score) shown in the regular chart reported for the same terms.

## Results

At the first step, in order to find the predicted target genes of miR-126, miRWalk 2.0 was implemented. Approximately 111 target genes were predicted to be the potential targets of miR-126. Moreover, 24 genes were also validated to be the target genes of miR-126. The predicted target could be the potential targets of miR-126 reported by miRWalk database. Checking DAVID database showed the involvement of miR-126 target genes in several signaling pathways indicated by the KEGG database including cancer pathway, neurotrophin signaling pathway, glioma, insulin signaling pathway, focal adhesion, chemokine signaling pathway and regulation of actin cytoskeleton (Table 1). Also, validated target genes of miR-126 were extracted from mirtarbase (Table 2) and predicted target genes of miR-126 were obtained from miRWalk 2.0 (Table 3).

Fisher’s exact test P values and enrichment scores [corresponding to false discovery rate (FDR)] were calculated to characterize the gene groups enriched in the target list. According to the results, *PIK3R2, KRAS, CRK* and *IRS1* were involved in the different pathways mentioned above and were targeted by miR-126. Furthermore, the target genes, were experimentally validated. 

**Fig.2:**
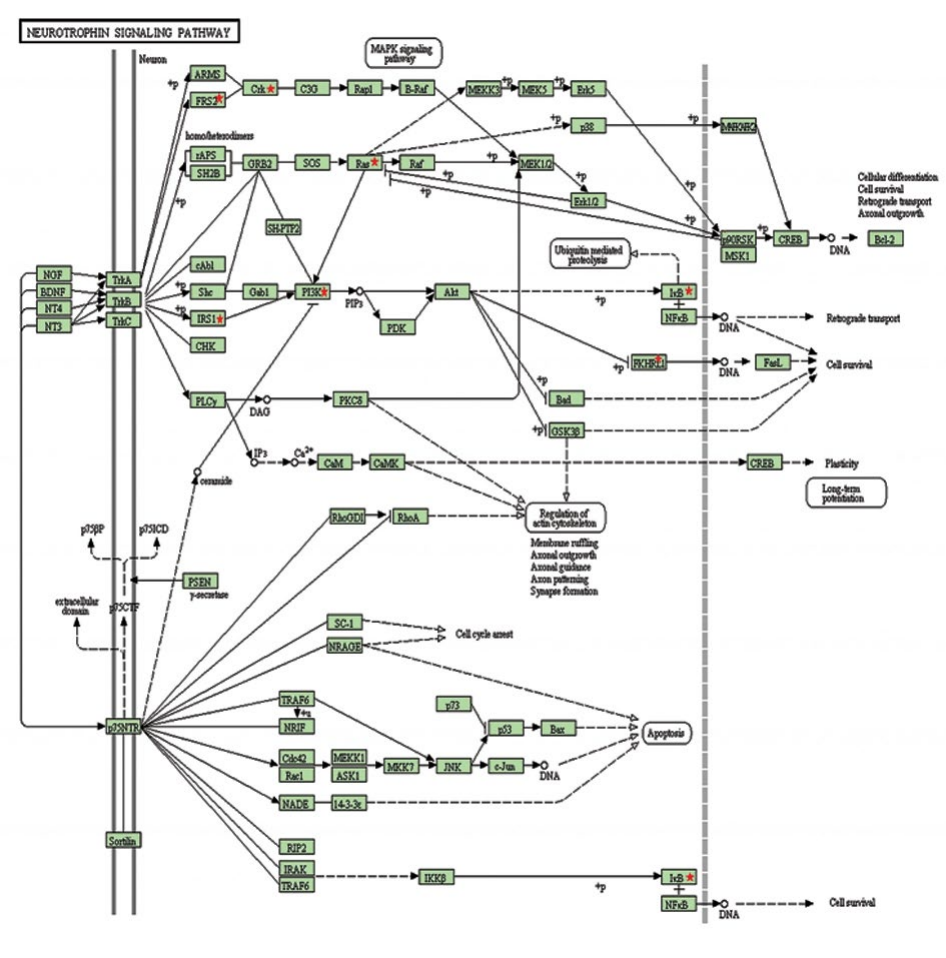
miR-126 is involved in neurotrophin signaling pathway pathways including IRS1, PI3K and IκB, which their partial diagram collected from KEGG pathway is demonstrated. Red stars mark targets of miR-126.

**Fig.3:**
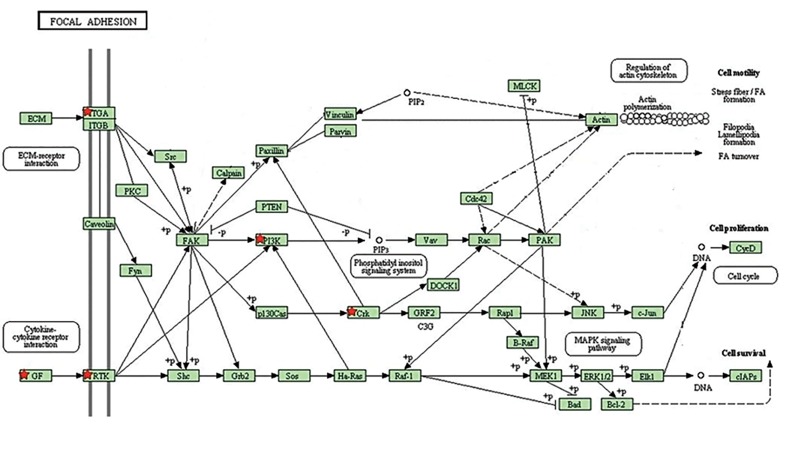
Involvement of miR-126 targetome in focal adhesion signaling pathway from KEGG is shown. miR-126 target genes are determined by red star marks.

Our evaluation determined that some KEGG pathways (Table 1) including neurotrophin (Fig .2) and focal adhesion signaling pathways (Fig .3) are the ones that are most statistically associated with miR 126 targeting. Interestingly, four genes namely, *KRAS, PIK3R2, IRS1* and *CRK* were influenced by miR 126. Among them, *PIK3R2* and *KRAS* were shown to be the most effective genes in focal adhesion and neurotrophin pathways. 

**Table 1:** The KEGG pathways reported by DAVID, concerning miR-126 targets based on TarBase 6.0


Signaling pathway	Count	%	P value	FDR	Genes

Neurotrophin signaling pathway	8	6.15	0.000035	0.036985	1398, 10818, 2309, 3667, 3845, 8660, 4792, 5296
Insulin signaling pathway	6	4.61	0.0034	3.5688779	1398, 7248, 3667, 3845, 8660, 5296
Aldosterone-regulated sodium reabsorption	4	3.07	0.0036	3.798433	3667, 3845, 8660, 5296
Prostate cancer	7	5.38	0.00005	0.052897	3480, 9134, 1869, 3845, 1027, 4792, 5296
Chronic myeloid leukemia	6	4.61	0.00024	0.252281	1398, 1869, 3845, 1027, 4792, 5296
Small cell lung cancer	6	4.61	0.0004	0.428764	9134, 1869, 1027, 4792, 3655, 5296
Pathways in cancer	10	7.69	0.00065	0.691123	1398, 3480, 9134, 1869, 7422, 3845, 1027, 4792, 3655, 5296
Chemokine signaling pathway	7	5.38	0.0027	2.838854	1398, 6387, 2309, 7852, 3845, 4792, 5296
Non-small cell lung cancer	4	3.07	0.0079	8.08554	1869, 2309, 3845, 5296
Glioma	4	3.07	0.012	12.1033	3480, 1869, 3845, 5296
Renal cell carcinoma	4	3.07	0.016	15.78153	1398, 7422, 3845, 5296
Melanoma	4	3.07	0.017	16.34347	3480, 1869, 3845, 5296
Pancreatic cancer	4	3.07	0.017	16.91404	1869, 7422, 3845, 5296
ErbB signaling pathway	4	3.07	0.028	26.3737	1398, 3845, 1027, 5296
Type II diabetes mellitus	3	2.3	0.05	41.96915	3667, 8660, 5296
Focal adhesion	5	3.84	0.066	51.80995	1398, 3480, 7422, 3655, 5296
Endometrial cancer	3	2.3	0.066	48.06802	2309, 3845, 5296
Colorectal cancer	3	2.3	0.14	78.64442	3480, 3845, 5296
Progesterone-mediated oocyte maturation	3	2.3	0.14	79.97993	3480, 3845, 5296
Regulation of actin cytoskeleton	4	3.07	0.23	93.51759	1398, 3845, 3655, 5296


FDR; False discovery rate. miR-126 target genes in several signaling pathways such as pathways in cancer, neurotrophin signaling pathway, glioma, insulin signaling pathway, focal adhesion, chemokine signaling pathway and regulation of actin cytoskeleton, etc.

**Table 2:** Targets of hsa-miR-126 validated by mirTarbase


Hsa-miR-126	Enterz gene ID	Brain^*^	Glioma^*^

SPRED1	161742	36	37
PLK2	10769	388	121
CCNE2	9134	27	37
RGS3	5998	69	74
TOM1	10043	178	102
CRK	1398	83	83
VEGFA	7422	52	158
PIK3R2	5296	70	279
VCAM1	7412	20	0
IRS1	3667	11	27
E2F1	1869	18	55
SOX2	6657	70	429
TWF1	5756	38	55
TWF2	11344	51	74
DNMT1	1786	48	74
KRAS	3845	32	9
IGFBP2	3485	84	457
PITPNC1	26207	34	46
MERTK	10461	12	46
EGFL7	51162	20	37
SLC7A5	8140	48	65
TEK	7010	40	0
ADAM9	8754	21	37
CXCL12	6387	44	0
FOXO3	2309	80	121
CXCR4	7852	34	27
CD97	976	52	83
TCF4	6925	187	93
Cdkn1b	1027	173	46
RHOU	58480	58	93
LRP6	4040	19	9
ADM	133	42	158
NFKBIA	4792	48	167


*; Transcripts per million (TPM).

**Table 3:** A part of targets of hsa-miR-126 predicted by mirWalk2


Gene	EntrezID	Brain^*^	Glioma^*^

PTPN9	5780	27	93
SOX2	6657	70	429
GOLPH3	64083	119	111
PEX5	5830	73	55
RGS3	5998	69	74
UBQLN2	29978	57	102
SPRED1	161742	36	37
CRK	1398	48	37
ITGA6	3655	31	27
IRS1	3667	11	27
LRP6	4040	19	9
SLC7A5	8140	48	65
HERPUD1	9709	86	167
PLK2	10769	388	121
SOX21	11166	13	74
GATAD2B	57459	32	18
ORMDL3	94103	37	83
ANTXR2	118429	25	0
PKD2	5311	21	9
PLXNB2	23654	64	74
FOXO3	2309	80	121
IGF1R	3480	36	102
QDPR	5860	374	65
SDC2	6383	115	55
IRS2	8660	37	121
LARGE	9215	39	46
TOM1	10043	178	102
MGEA5	10724	182	83


*; Transcripts per million (TPM).

## Discussion

So far, MiR-126 is the only characterized miRNA that shows endothelial cell (EC)-specific expression and the first vascular miRNA that was knocked out in mice. Loss- of-function studies in mice and zebrafish showed that miR126 possesses an important function in controlling vascular integrity and angiogenesis (17, 18). Previous studies indicated truncation of miR-126 in mouse as a pathogenic cause of leaky vessels, hemorrhage, and embryonic death, due to loss of vascular integrity and defective angiogenesis. *miR-126^-/-^* mice demonstrated severely hindered vascularization during development of cranial vessel and retina. In addition, *miR-126^-/-^* showed defective angiogenesis in response to angiogenic factors (19). 

Knockdown of miR-126 in zebrafish led to hemorrhage, and collapse of the dorsal aorta and primary cardinal veins, indicating a conserved function of miR-126. In vivo functional studies in mice and zebrafish showed a function for *Egfl7*(as the host gene of miR-126) in EC migration and vasculogenesis (20, 21). Interestingly, edema, defective cranial vessel and retina vascularization were the signs for *miR-126^-/-^* mice as well as *Egfl7^-^* knockout mice. The molecule responsible for the observed phenotype has been discussed. However, in *miR-126^-/-^* mice, *Egfl7* expression was not altered (21). Nonetheless, miR-126 expression in *Egfl7^-^* knockout mice was not reported (22). Recently, floxed alleles of *Egfl7 (Egfl7^Δ^)* and miR-126 (*miR-126^Δ^*) were generated (23). 

*Egfl7^Δ/Δ^* mice, in which miR-126 expression is not affected, are phenotypically normal whereas *miR-126^Δ/Δ^* mice, in which *Egfl7* is normally expressed, exhibit numerous previously explained embryonic and postnatal vascular phenotypes observed in *Egfl7*-knockout mice. These results indicated that miR-126 is an essential factor not only for angiogenesis but also for maintenance of vascular integrity. The in vivo functions of Egfl7 might be supported by its paralog Egfl8. This debate highlights the significance of minimally disruptive gene-targeting strategies because of the presence of intronic miRNAs in the genome. Neoangiogenesis is important for vascular regeneration in response to injury, such as myocardial infarction (MI).

*miR-126^-/-^* mice also exhibited reduced survival and defective cardiac neovascularization following MI, suggesting a significant role for miR-126 in neoangiogenesis (24). Previous reports have indicated that proangiogenic function of miR-126 is exerted through enhancement of MAP kinase and PI3K signaling in response to vascular endothelial growth factor (VEGF) and fibroblast growth factors (FGF) (20). Also, Saito et al. (25) examined alterations in epigenetic and expression levels of miR-126 in human cancer cells. Therefore, it seems that epigenetic modifications may control the expression of miR-126 as a tumor suppressor intronic miRNA through directly controlling the host target gene, EGFL7 (26). Apart from Spred-1, PIK3R2 and EGFL7, miR-126 targets vascular cell adhesion protein 1 (VCAM-1), thereby affects the regulation of the adhesion of leukocytes to the endothelium (27) defining another role for miR-126 in vascular inflammation. It was also reported that MiR-126 prevents tumorigenesis and is downregulated in many cancer lines (25, 26, 28).

Importantly, PIK3R2 and CT10 regulator of kinase (CRK) showed to be the targets for miR-126 in cancer cells (28). Evidence showing that PIK3R2 represses PI3KAKT signaling in ECs but enhances PI3K-AKT signaling in cancer cells, is not sufficient. Nevertheless, these results indicate that miR-126 is a multi-functional miRNA with important roles in angiogenesis, tumor growth and invasion, and vascular inflammation (29). Another target of miR-126 was revealed by Zhang et al. (10). They showed that miR-126 prevented cell cycle progression in breast cancer cells via targeting IRS-1 at 3'-UTR (8). Also, miR-126 targets *HOXA9*; the overexpression of *HOXA9* is associated with poor prognosis in acute myelogenous leukemia (26). These studies showed that miR-126 plays a significant role in tumorigenesis, tumor progression and metastasis (22, 28). 

Neurotrophins form a family of growth factors which play significant roles in neuronal development. This family has four members in mammals: i. Brain-derived neurotrophic factor (BDNF), ii. Nerve growth factor (NGF), iii. Neurotrophin-3 (NT3), and iv. Neurotrophin-4/5 (NT4/5) (30). Neurotrophins are synthesized as larger precursors which undertake proteolytical cleavage for production of mature neurotrophins. These factors perform multiple functions in the nervous system. As they may raise survival, differentiation, axon outgrowth or apoptosis (31, 32).

Barker indicated that dependingon the type of neurotrophin and the expression pattern of neurotrophin receptors (NTRs), neurotrophin is triggered by binding to two different classes of cell surface receptors namely, p75NTR and Trk receptors. The Trk receptors (TrkA, TrkB, and TrkC) comprise a large family of receptor tyrosine kinases, whereas p75NTR is structurally a member of the Fas/TNF-R family (33, 34). In primary cultures of rodent superior cervical ganglion sympathetic neurones, the TrkA ligands, NGF and NT3 enhance survival and neurite outgrowth during embryonic development, whereas the p75NTR ligand, BDNF has been reported to induce apoptosis (35). Evangelopoulos et al. (36) showed that TrkB-Fc or TrkC-Fc receptors are useful tools for modification of the survival of neuroblastoma cells.

Ahn et al. (11) showed that glioma invasion mediated by the p75 neurotrophin receptor (p75NTR/CD271), requires regulated interaction with PDLIM1 (a member of the ALP subfamily of PDZ/LIM proteins). Wadhwa et al. (37) suggested that Trk A and Trk B are involved in early stages of tumor pathogenesis, glial proliferation, and progression of malignancy. Lawn et al. (38) and Forsyth et al. (39) proved that neurotrophin signaling pathway promotes the growth and proliferation of glioma cell line. Li et al. (40) confirmed that different mechanisms found for regulations of NDAP (neurotrophin-regulated neuronal development-associated protein) expression by neurotrophins, might be checkpoints for apoptosis during neuronal development. 

## Conclusion

Our results suggested a significant role for neurotrophin signaling pathway in gliomagenesis. According to our findings, overexpression of miR-126 may lead to downregulation of IRS-1 and subsequently formation of glioma cancer stem cell.

## References

[1] Yin Y, Qiu S, Peng Y (2016). Functional roles of enhancer of zeste homolog 2 in gliomas. Gene.

[2] Di Leva G, Garofalo M, Croce CM (2014). MicroRNAs in cancer. Annu Rev Pathol.

[3] Kala R, Peek GW, Hardy TM, Tollefsbol TO (2013). MicroRNAs: an emerging science in cancer epigenetics. J Clin Bioinforma.

[4] Cullen BR (2009). Viral and cellular messenger RNA targets of viral microRNAs. Nature.

[5] Grady WM, Tewari M (2010). The next thing in prognostic molecular markers: microRNA signatures of cancer. Gut.

[6] Dolecek TA, Propp JM, Stroup NE, Kruchko C (2012). CBTRUS statistical report: primary brain and central nervous system tumors diagnosed in the United States in 2005-2009. Neuro Oncol.

[7] Musiyenko A, Bitko V, Barik S (2008). Ectopic expression of miR-126*, an intronic product of the vascular endothelial EGF-like 7 gene, regulates prostein translation and invasiveness of prostate cancer LNCaP cells. J Mol Med (Berl).

[8] Meister J, Schmidt MHH (2010). miR-126 and miR-126*: new players in cancer. ScientificWorldJournal.

[9] Sun YQ, Zhang F, Bai YF, Guo LL (2010). miR-126 modulates the expression of epidermal growth factor-like domain 7 in human umbilical vein endothelial cells in vitro. Nan Fang Yi Ke Da Xue Xue Bao.

[10] Zhang J, Du YY, Lin YF, Chen YT, Yang L, Wang HJ (2008). The cell growth suppressor, mir-126, targets IRS-1. Biochem Biophys Res Commun.

[11] Ahn BY, Saldanha-Gama RF, Rahn JJ, Hao X, Zhang J, Dang NH (2016). Glioma invasion mediated by the p75 neurotrophin receptor (p75(NTR)/CD271) requires regulated interaction with PDLIM1. Oncogene.

[12] Kuruvilla R, Ye H, Ginty DD (2000). Spatially and functionally distinct roles of the PI3-K effector pathway during NGF signaling in sympathetic neurons. Neuron.

[13] Borggrefe T, Lauth M, Zwijsen A, Huylebroeck D, Oswald F, Giaimo BD (2016). The notch intracellular domain integrates signals from Wnt, Hedgehog, TGFβ/BMP and hypoxia pathways. Biochim Biophys Acta.

[14] Tidyman WE, Rauen KA (2009). The RASopathies: developmental syndromes of Ras/MAPK pathway dysregulation. Curr Opin Genet Dev.

[15] Park DM, Rich JN (2009). Biology of glioma cancer stem cells. Mol Cells.

[16] Luan Y, Zuo L, Zhang S, Wang G, Peng T (2015). MicroRNA-126 acts as a tumor suppressor in glioma cells by targeting insulin receptor substrate 1 (IRS-1). Int J Clin Exp Pathol.

[17] Salajegheh A, Pakneshan S, Rahman A, Dolan-Evans E, Zhang S, Kwong E (2013). Co-regulatory potential of vascular endothelial growth factor-A and vascular endothelial growth factor-C in thyroid carcinoma. Hum Pathol.

[18] Ebrahimi F, Gopalan V, Smith RA, Lam AKY (2014). MiR-126 in human cancers: clinical roles and current perspectives. Exp Mol Pathol.

[19] Hsu SD, Tseng YT, Shrestha S, Lin YL, Khaleel A, Chou CH (2014). MiRTarBase update 2014: an information resource for experimentally validated miRNA-target interactions. Nucleic Acids Res.

[20] Huang DW, Sherman BT, Lempicki R a (2009). Systematic and integrative analysis of large gene lists using DAVID bioinformatics resources. Nat Protoc.

[21] Huang DW, Sherman BT, Lempicki R a (2014). Bioinformatics enrichment tools: Paths toward the comprehensive functional analysis of large gene lists. Nucleic Acids Res.

[22] Schmidt M, Paes K, De Mazière A, Smyczek T, Yang S, Gray A (2007). EGFL7 regulates the collective migration of endothelial cells by restricting their spatial distribution. Development.

[23] Kuhnert F, Mancuso MR, Hampton J, Stankunas K, Asano T, Chen CZ (2008). Attribution of vascular phenotypes of the murine Egfl7 locus to the microRNA miR-126. Development.

[24] Wang S, Aurora AB, Johnson BA, Qi X, McAnally J, Hill JA (2008). The endothelial-specific microrNA miR-126 governs vascular integrity and angiogenesis. Dev Cell.

[25] Saito Y, Friedman JM, Chihara Y, Egger G, Chuang JC, Liang G (2009). Epigenetic therapy upregulates the tumor suppressor microRNA-126 and its host gene EGFL7 in human cancer cells. Biochem Biophys Res Commun.

[26] Sun Y, Bai Y, Zhang F, Wang Y, Guo Y, Guo L (2010). miR-126 inhibits non-small cell lung cancer cells proliferation by targeting EGFL7. Biochem Biophys Res Commun.

[27] Harris TA, Yamakuchi M, Ferlito M, Mendell JT, Lowenstein CJ (2008). MicroRNA-126 regulates endothelial expression of vascular cell adhesion molecule 1. Proc Natl Acad Sci USA.

[28] De Mazière A, Parker L, Van Dijk S, Ye W, Klumperman J (2008). Egfl7 knockdown causes defects in the extension and junctional arrangements of endothelial cells during zebrafish vasculogenesis. Dev Dyn.

[29] Wang S, Olson EN (2009). AngiomiRs-key regulators of angiogenesis. Curr Opin Genet Dev.

[30] Bothwell M (1995). Functional interactions of neurotrophins and neurotrophin receptors. Annu Rev Neurosci.

[31] Huang EJ, Reichardt LF (2001). Neurotrophins: roles in neuronal development and function. Annu Rev Neurosci.

[32] Sofroniew MV, Howe CL, Mobley WC (2001). Nerve growth factor signaling, neuroprotection, and neural repair. Annu Rev Neurosci.

[33] Barker PA (1998). p75NTR: a study in contrasts. Cell Death Differ.

[34] Chao M V (1994). The p75 neurotrophin receptor. J Neurobiol.

[35] Francis NJ, Landis SC (1999). Cellular and molecular determinants of sympathetic neuron development. Annu Rev Neurosci.

[36] Evangelopoulos ME, Weis J, Kruttgen A (2004). Neurotrophin effects on neuroblastoma cells: correlation with trk and p75NTR expression and influence of Trk receptor bodies.J Neurooncol. Springer.

[37] Wadhwa S, Nag TC, Jindal A, Kushwaha R, Mahapatra AK, Sarkar C (2003). Expression of the neurotrophin receptors Trk A and Trk B in adult human astrocytoma and glioblastoma. J Biosci.

[38] Lawn S, Krishna N, Pisklakova A, Qu X, Fenstermacher DA, Fournier M (2015). Neurotrophin signaling via TrkB and TrkC receptors promotes the growth of brain tumor-initiating cells. J Biol Chem.

[39] Forsyth PA, Krishna N, Lawn S, Valadez JG, Qu X, Fenstermacher DA (2014). p75 neurotrophin receptor cleavage by α-and γ-secretases is required for neurotrophin-mediated proliferation of brain tumorinitiating cells. J Biol Chem.

[40] Li HL, Li Z, Qin LY, Liu S, Lau LT, Han JS (2006). The novel neurotrophin-regulated neuronal development-associated protein, NDAP, mediates apoptosis. FEBS Lett.

